# More weighted cancellous bone can be harvested from the proximal tibia with less donor site pain than anterior iliac crest corticocancellous bone harvesting: retrospective review

**DOI:** 10.1186/s13018-021-02364-y

**Published:** 2021-03-26

**Authors:** Hanju Kim, Ajit Kumar Kar, Aditya Kaja, Eic Ju Lim, Wonseok Choi, Whee Sung Son, Jong-Keon Oh, Seungyeob Sakong, Jae-Woo Cho

**Affiliations:** 1 Department of Orthopaedic Surgery, Korea University Guro Hospital, Korea University Medicine, 148, Gurodong-ro, Guro-gu, Seoul, 08308 South Korea; 2 Department of Orthopedic Surgery, Shanti memorial Hospital, Cuttack, India; 3 Department of Orthopedic Surgery, James Cook University Hospital, Middlesbrough, UK

**Keywords:** Autogenous bone graft, Harvesting donor site, Iliac crest vs Proximal tibia, Harvesting amounts, Donor site pain

## Abstract

**Background:**

Iliac crest is the most preferred autogenous bone graft harvesting donor site while it has sorts of complications like prolonged pain, hematoma, and fracture. Harvesting cancellous bone from proximal tibia is also increasingly being used because of lower complications and less donor site pain. However, there are lack of studies to compare these two donor sites in detail. Thus, we proposed to investigate the available amount of autogenous bone graft from the proximal tibia.

**Methods:**

Fifty-one patients who underwent simultaneous bone graft harvest from the PT and the AIC to fill up the given critical sized bone defects were enrolled in this study. We prospectively collected data including the weight of the harvested bone, donor site pain using the visual analog scale (VAS) score, and complications between the two sites.

**Results:**

The mean weight of cancellous bone harvested from the PT was greater than AIC (33.2g vs. 27.4g, *p* = 0.001). The mean VAS score was significantly lesser in the PT up to 60 days after harvesting (*p* < 0.001). There was persistent pain up to 90 days in four PT patients and in seven AIC patients. The major complication was reported only in AIC patients (11.8%).

**Conclusions:**

Harvesting cancellous bone from the PT is an acceptable alternative to the AIC for autogenous bone grafting owing to availability of more weighted graft bone and less donor site pain.

## Introduction

Bone grafting has played a vital role in orthopedic surgery owing to its use in treating atrophic nonunion, reconstruction of large bone defect nonunion, and in musculoskeletal infection, trauma, and tumor [1, 2]. There are several options for graft selection: autogenous or allogenous, cancellous or cortical bone, demineralized bone matrix, calcium phosphate-based bone graft substitute, or autogenous bone marrow [3, 4]. Although the abovementioned options are all widely accepted in orthopedic surgery, autogenous bone grafts remain the gold standard as they exhibit the desirable qualities of osteoinduction, osteoconduction, and osteogenesis with minimal adverse reactions [5, 6].

Autogenous bone can be harvested from various donor sites such as the iliac crest (IC), proximal tibia (PT), distal tibia, distal radius, and the olecranon process [5]. Among these sites, the IC has been regarded as the first priority source and is mostly used in the clinical setting, as it provides easy access to good quality and quantity cancellous autograft [2]. However, significant complications have been reported such as prolonged pain, hematoma, seroma, deep infection, fracture, visceral complications, and paresthesia [7–10]. Major donor site complication rates of 2.4 to 6.2% have been reported in studies on the IC graft, thus mandating the need for alternative donor sites [11, 12].

Harvesting cancellous bone from the PT was first introduced by O’Keeffe et al. [13] and has gained popularity over time. The advantages of harvesting bone from the PT include ease of access to the involved limb, lack of need for a separate prepping and draping site for treating the ipsilateral limb, comparable volume, and lower complication rates [5]. Recent studies reported less donor site pain in the PT compared to bone graft harvesting from the anterior IC (AIC) or the PT [14–16]. None of these studies compared morbidity and quantity of bone harvest in a single individual, and they failed to compare the donor site pain while maintaining ideal matching. We believe that this limits the understanding of the outcomes reported previously, considering the subjectiveness of pain perception and the different bone densities among patients. Thus, there is a constant debate on the ideal graft harvesting site, and no clear conclusion exists.

Recently, translational medicine (TM) is an emerging method and process of facilitating medical advances efficiently by translation result from clinical studies into everyday clinical practice [17, 18]. In the context of translational orthopedics, this study is designed to help clinicians determine the favorable site of bone harvesting in donor site pain and available amount.

This study aimed to analyze the pain level of autogenous bone graft from the PT and the AIC and compare the available amount of graft between both sites in patients who underwent bone harvesting simultaneously.

## Material and methods

### Design and setting

This was a retrospective review of prospectively collected case series at single level 1-trauma centers, which was approved by our institutional review board. The data were collected prospectively between June 2015 and February 2018. All consecutive patients who required autogenous bone grafting were enrolled in the initial cohort. Patients who had undergone a single site of bone harvest, had a previous history of bone harvest surgery, or had an incomplete bone harvest procedure were excluded. Patients who underwent bone harvesting from the PT and the IC simultaneously for treating critical-sized bone defects were included in the final cohort.

### Techniques of bone harvesting (PT and AIC)

**Fig. 1:**
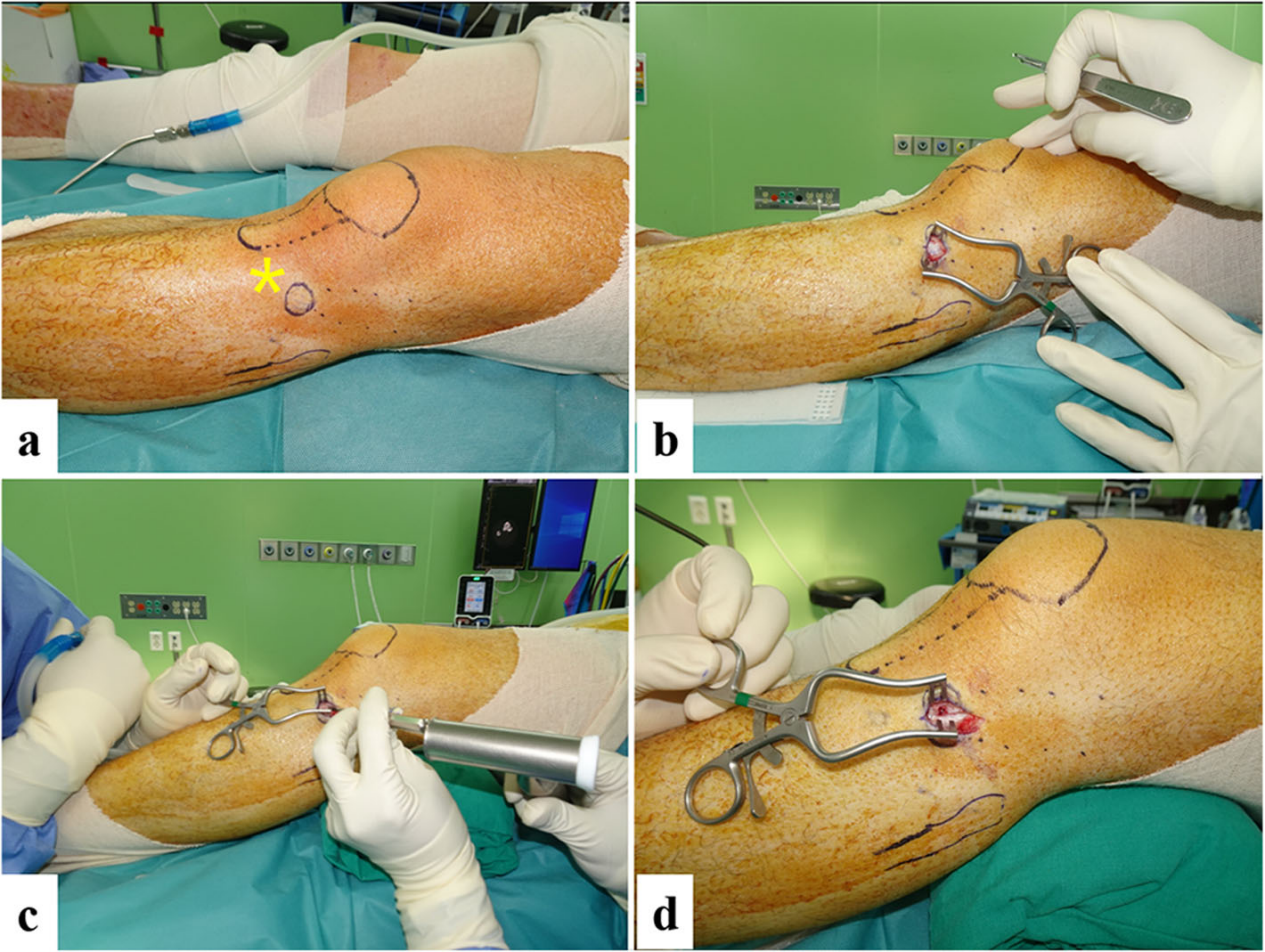
Bone harvesting from proximal tibia. **a** The patella, tibial tuberosity, fibular head, and Gerdy’s tubercle (yellow asterisk) were marked. **b** 2–3-cm linear skin incision over Gerdy’s tubercle. **c** Cortical bony window was created. **d** Cancellous bone was scooped out

All surgeries were carried out by two senior orthopedic surgeons using a single technique of bone harvesting. After the induction of general anesthesia, patients were placed in a supine position on a radiolucent table with a bump under the ipsilateral buttock to elevate the hemi pelvis, align the lower limb, and keep it maintained internally rotated during the procedure. In the PT group, using a sterile marker, the lateral tibio-femoral joint line, the tibial tuberosity, the fibular head, and Gerdy’s tubercle were marked. The harvest was centered over the Gerdy’s tubercle. A 2–3-cm linear incision was directed superolaterally to inferomedially through the skin and subcutaneous tissue directly over Gerdy’s tubercle. This was continued through the skin and subcutaneous tissue down to the periosteum, then retracted to expose the bony prominence. Cortical bone was osteotomized using a one-quarter inch osteotome size 1 cm × 1 cm. A self-retractor was placed, and the bone graft was carefully scooped out using a straight and curved curette till a complete harvest was done. A safety margin of the epiphyseal line of the proximal tibia was set to avoid an iatrogenic fracture of the harvest site (Fig. 1). Joint motion of the bone harvesting site was permitted immediately after surgery. Toe touch partial weight bearing was started from the day after surgery, and the patients were instructed to bear their full weight at one month after bone harvesting.

**Fig. 2:**
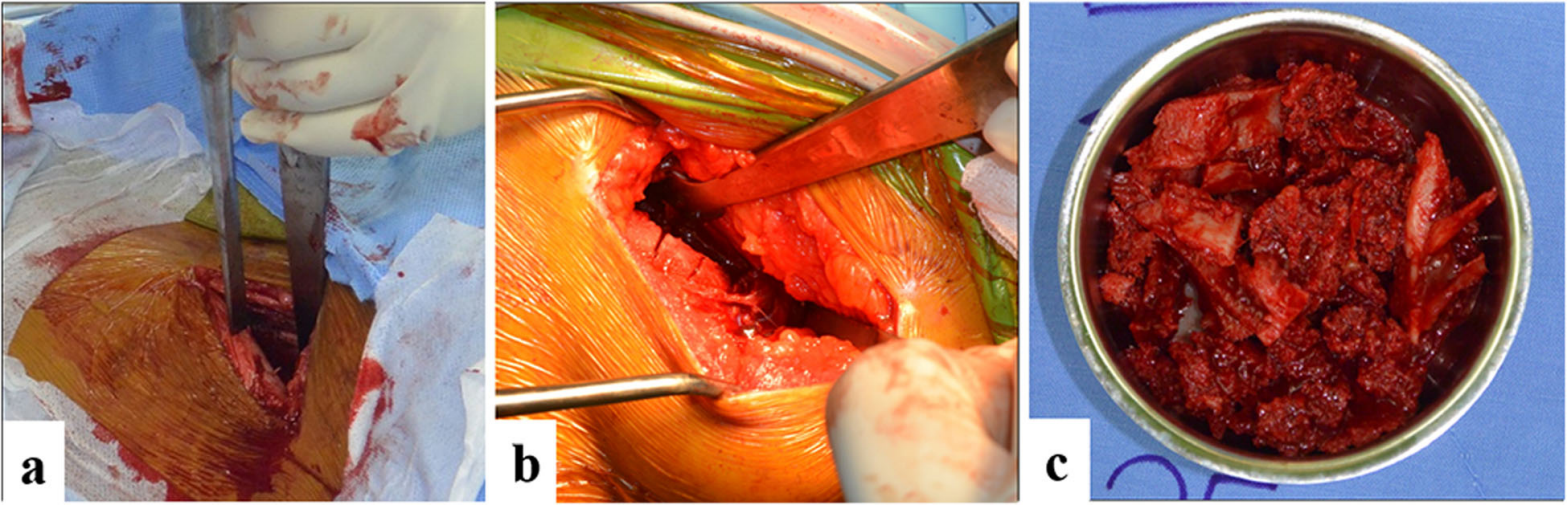
Bone harvesting from AIC. **a** 5-cm skin incision along IC, 1.5 cm posterior to the anterior superior iliac spine. After elevation of iliacus, osteotomies of the inner table (**b**) leaving one third of lateral cortex intact. **c** Corticocancellous bone was harvested

For bone harvesting from the AIC, a 5-cm skin incision was taken approximately 1.5 cm posterior to the anterior superior iliac spine to avoid injury to the lateral femoral cutaneous nerve. The intermuscular interval between the external oblique abdominis and the gluteus muscle was developed, and careful dissection was made to expose the superior aspect of the IC. The iliacus, muscle which was attached at the inner surface of the iliac fossa, was elevated sharply using a Cobb’s retractor to expose the inner table of the iliac bone. Osteotomies along the IC were made approximately at two thirds of the width toward the medial aspect to prevent perforating the lateral cortex, leaving one third of the lateral cortex intact. The corticocancellous bone was then harvested using curved osteotome and curved curette. After surgery, the patients were permitted to bear their weight depending on the persistence of pain (Fig. 2).

NSAIDs was used for postoperative pain control as the basis. In cases of unavailable using NSAIDs due to medical problems, tramadol was used instead.

### Measurement of the weight of the harvested bone

**Fig. 3:**
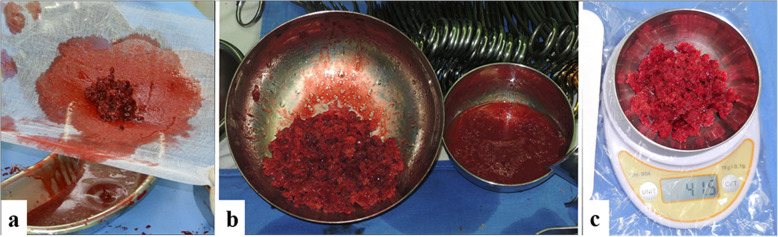
Intraoperative measurement of the weight of harvested bone. **a** The harvested bone was placed on a sterile gauze and compressed. **b** Blood and fat were separated from the cancellous bone. **c** The weight was measured by electronic weighing scale

Intra-operatively, the weight of harvested bone from both sites were measured using a uniform method with an electronic weighing scale. The harvested bone from both sites were each placed on a separate sterile gauze and compressed to release blood. They were then placed on a pre-weighed sterile bowl and measured independently. The data were entered into the operative records (Fig. 3).

### Donor site pain evaluation

The perioperative and postoperative records of the patients enrolled in the study were compiled into a uniform case report form (CRF) by an independent research investigator. We used the visual analog scale (VAS) for quantification of pain. The day after surgery, an independent research investigator provided two different sheets of the VAS questionnaire to each patient, one for each site of harvest. The scores were entered into the CRF and this process was carried out from postoperative day 1 (POD1) to postoperative day 7 (POD7) during the in-hospital stay and evaluated at the regular outpatient visits at 2 weeks (POD14), 1 month (POD30), 2 months (POD60), 3 months (POD90), and 6 months (POD180).

### Complications associated with bone harvesting

Plain radiographs of the pelvis and the knee were routinely evaluated at postoperative day 0 and during regular follow-up visits. Early postoperative complications such as iatrogenic fractures, nerve injury, hematoma, and wound complications were evaluated during the in-hospital stay. Late postoperative complications such as deep infection at the harvest site, signs of nerve injury, and delayed fractures were clinically examined during follow up visits.

### Statistical analysis

Statistical analysis was conducted using the Statistical Package for the Social Sciences version 23.0 (SPSS Inc., Chicago, USA). The paired *T* test was performed to determine significant differences of weight of the harvested bone between PT and AIC. The Wilcoxon signed-rank test was performed to determine the differences of VAS between PT and AIC.

## Results

Of a total of 167 autogenous bone grafts harvested from 106 patients, 61 were from the AIC, four from the posterior IC, and 102 were from the PT. The final cohort consisted of 51 patients who met the criteria and underwent simultaneous bone graft harvest from the AIC and the PT to fill up the given bony defects. There were 40 men and 11 women with an average age of 48 years (range, 19–75 years). The average BMI was 25.3 kg/m^2^ (range, 19.5–33.4 kg/m^2^). All bony defects were of the segmental type in the diaphysis of long bones with an average defect length of 8.6 cm ± 4.8 cm. The bony defects resulted from posttraumatic osteomyelitis in 34 cases, infected nonunion in 15 cases, and open fracture in two cases (Table 1).

**Table 1:** Patient characteristics

Demographic characteristic	
Patients, *n*	51
Sex, M/F	40/11
Average age (range), yr	48.2 (19–75)
BMI (range), kg/m^2^	25.3 (19.5–33.4)
Reasons of bony defect, *n* (%)	
Posttraumatic osteomyelitis	34 (66.7)
Infected nonunion	15 (29.4)
Open fracture	2 (3.9)
Length of bony defect (range) cm	8.6 (3.6–20.9)

The mean weight of cancellous bone harvested from the PT was 33.2 g ± 10.0g. The average weight of corticocancellous bone harvested from the AIC was 27.4 g ± 9.4g. There was a statistical significant difference among the 2 groups (*p* = 0.001).

**Fig 4:**
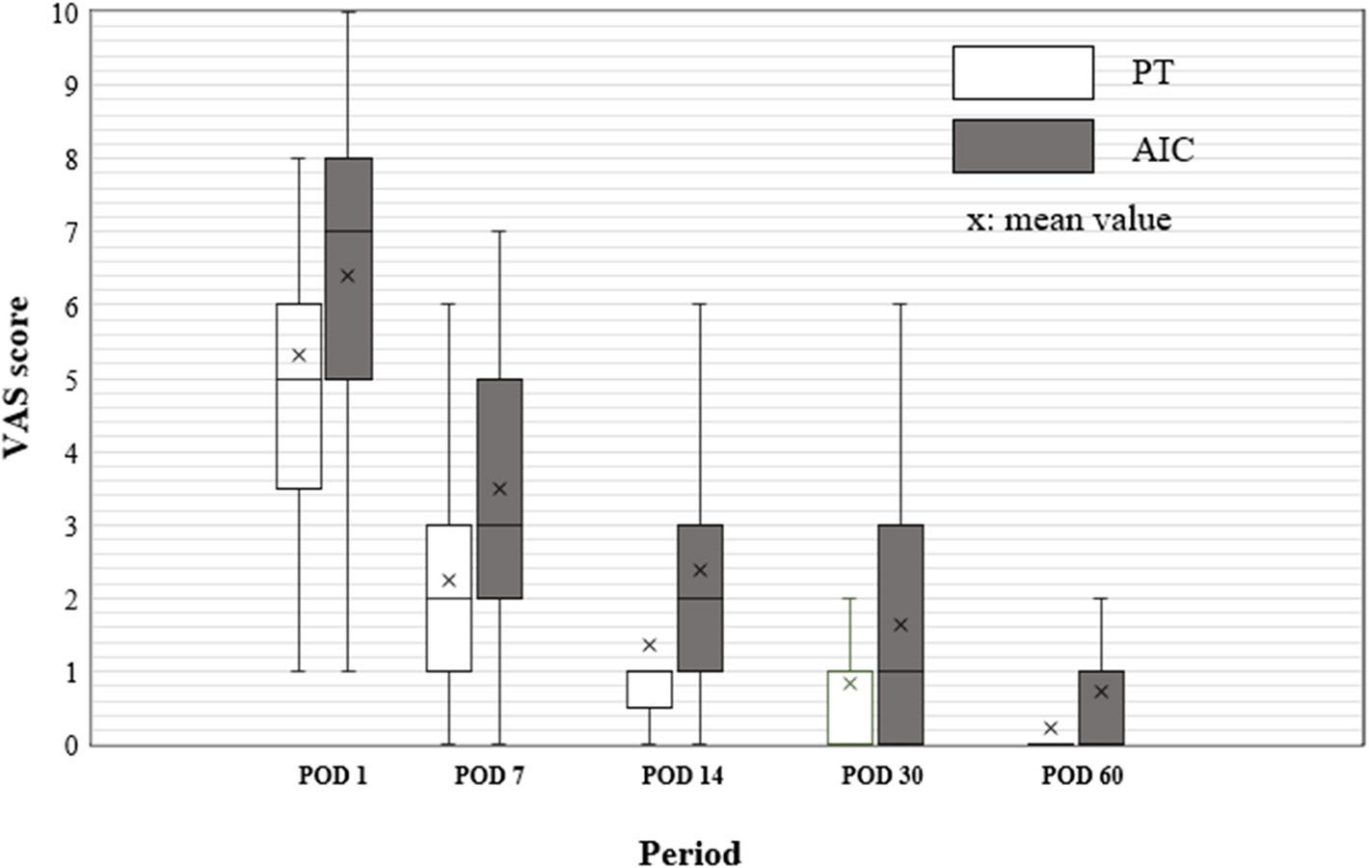
Comparison of donor site pain between PT and AIC. The mean VAS scores for the AIC harvesting site on POD1, POD7, POD14, POD30, and POD60 were significantly higher than those for the PT (*p* < 0.001)

The mean VAS scores for the PT harvesting site on POD1, POD7, POD14, POD30, POD60, and POD 90 were 5.33, 2.25, 1.37, 0.82, 0.24, and 0.08, respectively. The mean VAS scores for the AIC harvesting site on POD1, POD7, POD14, POD30, POD60, and POD 90 were 6.39, 3.49, 2.39, 1.63, 0.73, and 0.16, respectively. The mean VAS scores from POD1 to POD60 in the AIC group were significantly higher than those for the PT (p < 0.001) (Fig. 4). However, there was no statistically significant difference in the VAS scores on POD 90 (*p* = 0.103). Persistence of pain was observed in the PT group, identified in 24, eight, and four patients at POD30, POD60, and POD90, respectively. The pain in the AIC bone harvesting group was identified in 35, 21, and seven patients at POD30, POD60, and POD90, respectively (Table 2).

**Table 2:** Overall results and comparison according to harvesting site

	PT	AIC	*P* value
Weight of harvested bone, g	33.2 ±12.1	27.4 ± 9.4	0.001
Harvested bone type	Cancellous bone	Corticocancellous bone	<0.001
Donor site pain			
VAS-POD1, (*n*)	5.33 ± 2.22, (51)	6.39 ± 2.25, (51)	<0.001
VAS-POD7, (*n*)	2.25 ± 1.75, (47)	3.49 ± 1.92, (48)	<0.001
VAS-POD14, (*n*)	1.37 ± 1.57, (38)	2.39 ± 1.81, (45)	<0.001
VAS-POD30, (*n*)	0.82 ± 1.37, (24)	1.63 ± 1.56, (35)	<0.001
VAS-POD60, (*n*)	0.24 ± 0.62, (8)	0.73 ± 1.00, (21)	<0.001
VAS-POD90, (*n*)	0.08 ± 0.27, (4)	0.16 ± 0.42, (7)	0.103

Six major complications including one case of hematoma that needed incisional evacuation, two cases of paresthesia on the lateral aspect of the proximal thigh that were related with a lateral femoral cutaneous nerve injury, two cases of iliac wing fracture, and one case of surgical site infection were found in the AIC bone harvesting group. (6/51, 11.8%). However, there was no complication in the PT bone harvesting group.

## Discussion

The important finding in the present study was that more cancellous bone could be harvested from the PT than from the AIC with less donor site pain in a single subject. Approximately 30% more weighted graft could be obtained from the PT than the IC in the clinical setting of our study. This results varied slightly from previous cadaveric studies. Burk et al. reported that a larger graft volume could be harvested from the AIC than from the PT in a cadaver (20.58 ml from the AIC, 9.03 ml from the PT) [19]. Gerresen et al. concluded that approximately equal amounts of bone were available at both donor sites [20]. Other cadaveric studies reported contradictory results demonstrating that the PT yielded a significantly greater mean volume of compressed cancellous bone than the AIC [21]. An in vivo study of autogenous bone graft volume also demonstrated superior quantity from the PT in 16 patients when compared to the AIC in 12 patients. However, they failed to find a statistically significant comparable quantity between the PT and AIC groups [22]. Similar to this study, our in vivo result revealed that the PT allows more weighted cancellous bone harvesting than the AIC in a setting of automatically paired matching of age, sex, and bone mineral density and found a statistical difference between the two sites.

Donor site pain is one of the compromising morbidities after autogenous bone harvesting. In our study, the pain at the AIC site was more severe compared to that of PT with scores that were almost double at the end of POD14 and POD30. In addition, the pain at the AIC sites persisted until POD90 in 7 of 51 patients (13%). These findings are consistent with those in the literature. Baumhauer et al. prospectively compared patient-reported outcomes of acute and persistent pain in the harvesting site at 1 year after surgery. They reported that donor site pain at 3 weeks was more significant at the IC than the PT sites, and persistent pain in the harvesting site was observed for a year following foot and ankle surgery in some patients (8.5%) [23]. Mauffrey et al. also concluded that pain was more severe at the AIC site at 1 and 4 weeks postoperatively when compared to both the olecranon and PT sites [22]. Our finding can warrant previous results using different methods of eliminating the error in the VAS perception between subjects by comparing the VAS from two donor sites in a single subject.

Though autogenous bone grafting has proven its efficacy as a gold standard procedure for treating atrophic and defect nonunion acting as a biological scaffold and stimulator of bone formation [2–4, 24], various complications and morbidity associated with the bone harvesting procedure necessitate surgeons to select ideal harvesting sites or techniques with careful considerations. Compared to the relatively high rate of complications with IC bone grafts [7, 10, 25], published complication rates of PT donor sites have been low. O’Keeffe et al. reported a complication rate of 1.3% in 206 patients with 230 PT bone graft harvests including hematoma, tibial eminence fracture, and superficial surgical site infection [13]. Geideman et al. reported only four minor complications in 155 patients who underwent a foot and ankle procedure utilizing the ipsilateral PT as the donor site for autologous bone grafts. There was one case of harvesting site hematoma and three case of persistent pain at the donor site, which was more than those at the operative site [26].

The overall complication rate is low; however, fracture at the donor site is a considerably serious complication than that at other non-weight bearing donor sites. Kim et al. reported four tibial bone fractures in 105 patients [27]. Hughes and colleagues reported two harvesting fractures in 75 patients owing to performing exercise at 3 months and falls at 9 days after surgery, in which both fractures were treated conservatively. In our study, there were two cases of iliac wing fracture after AIC bone harvesting. Though patients complained of moderate donor site pain, the fractures were treated successfully without any long-term morbidity. However, there was no case of iatrogenic tibial fracture after PT bone harvesting.

The question of how much cancellous bone in proximal tibia should be left intact is left answered. Alt et al. concluded that the risk of postoperative fracture is not increased when 2 cm of cancellous bone remains at the plateau in a fresh cadaver study [28]. This study also set the safety margin of bone harvesting by leaving the cancellous bone above the epiphyseal line intact at the plateau. Although the minimal volume threshold at which the incidence of depression fracture occurs need to be further investigated, many researchers, in their clinical studies, have advocated that some amount of cancellous bone should be left intact at the plateau to avoid iatrogenic fractures [21, 29–31]. We could prevent the iatrogenic fracture by preserving the safety margin during harvesting the cancellous bone from the PT.

The limitations of this study include the fact that the amount of bone was compared by weight. Previous studies could measure the volume of harvested bone by measuring the displacement of fluid indirectly, in which when a known volume of water was placed into a 30-ml syringe in which the cancellous bone was located, the displacement could then represent the volume of the graft [21, 22]. We compared the weight of the grafting material because corticocancellous bone could not be measured using this method. Thus, we decided to compare the weight of the grafted bone after compression with a sterile gauze to eliminate fat and blood as much as possible. This method has not been validated by other studies and might lead to an error in calculation of the weight. However, we tried to minimize this error by applying unified methods in every case. Another limitation of this study is the lack of biochemical analysis of grafts. The future study which compare the osteogenic properties of harvested bone would be needed.

Additionally, the pain at the donor sites in each single subject was compared in this study. This unique cohort is different from that of previous studies. We used this method to reduce error from the subjective perception of the VAS, un-unified pain killer use, and patient demographic factors such as age, sex, and BMI. Though this could be the strong point of our study, a reciprocal masking effect of the pain in either of the two sites may be possible, which could make our comparison more significant.

## Conclusion

Harvesting cancellous bone from the PT is an acceptable alternative to the AIC for autogenous bone grafting, with significant more weighted harvested bone with lesser donor site’s pain. To find out the superiority of osteogenic capacity, the future biochemical analysis would be needed.

## Data Availability

The datasets used and/or analyzed during the current study are available from the corresponding author on reasonable request.
